# Early Aberrant Angiogenesis Due to Elastic Fiber Fragmentation in Aortic Valve Disease

**DOI:** 10.3390/jcdd8070075

**Published:** 2021-06-25

**Authors:** Robert B. Hinton, Amy L. Juraszek, Amy M. Opoka, Benjamin J. Landis, J. Michael Smith, Robert P. Mecham, Kevin E. Bove

**Affiliations:** 1 Divisions of Cardiology, Cincinnati Children’s Hospital Medical Center, Cincinnati, OH 45229, USA; amy.opoka@cchmc.org; 2 Pediatric Cardiology, Wolfson Children’s Hospital, Jacksonville, FL 32207, USA; juraszal@gmail.com; 3 Division of Pediatric Cardiology, Indiana University School of Medicine, Indianapolis, IN 46204, USA; benjland@iu.edu; 4 The Christ Hospital Heart Network, Cincinnati, OH 45219, USA; jmichaelsmith62@gmail.com; 5 Department of Cell Biology and Physiology, Washington University, St. Louis, MO 63130, USA; bmecham@wustl.edu; 6 Division of Pathology, Cincinnati Children’s Hospital Medical Center, Cincinnati, OH 45229, USA; kevin.bove@cchmc.org

**Keywords:** heart valves, aortic root, elastic fiber, angiogenesis, pediatrics

## Abstract

Elastic fiber fragmentation (EFF) is a hallmark of aortic valve disease (AVD), and neovascularization has been identified as a late finding related to inflammation. We sought to characterize the relationship between early EFF and aberrant angiogenesis. To examine disease progression, regional anatomy and pathology of aortic valve tissue were assessed using histochemistry, immunohistochemistry, and electron microscopy from early-onset (<40 yo) and late-onset (≥40 yo) non-syndromic AVD specimens. To assess the effects of EFF on early AVD processes, valve tissue from Williams and Marfan syndrome patients was also analyzed. Bicuspid aortic valve was more common in early-onset AVD, and cardiovascular comorbidities were more common in late-onset AVD. Early-onset AVD specimens demonstrated angiogenesis without inflammation or atherosclerosis. A distinct pattern of elastic fiber components surrounded early-onset AVD neovessels, including increased emilin-1 and decreased fibulin-5. Different types of EFF were present in Williams syndrome (WS) and Marfan syndrome (MFS) aortic valves; WS but not MFS aortic valves demonstrated angiogenesis. Aberrant angiogenesis occurs in early-onset AVD in the absence of inflammation, implicating EFF. Elucidation of underlying mechanisms may inform the development of new pharmacologic treatments.

## 1. Introduction

Aortic valve disease (AVD) obstructs outflow from the heart, affects 2.5% of the general population, and remains a significant cause of mortality [1,2]. Genetic and environmental risk factors have been identified. The treatment for AVD continues to be primarily surgical, restricted to late-stage disease, and the number of replacement procedures increases rapidly with the aging population [3,4]. A central problem in the field remains the need to better understand early AVD processes and alternative medical treatment strategies [5]. AVD is characterized by cell and matrix abnormalities that are well established. Advances in human genetics and developmental biology have elucidated mechanisms that contribute to pathogenesis [6,7], but to date, early disease processes remain poorly understood, and no pharmacologic-based treatments that directly treat AVD have been established.

Aberrant angiogenesis was originally described in cancer literature, and since then, a fundamental role for this biologic process has been established in other disease states that affect typically avascular tissue [8,9]. Aberrant angiogenesis has been described in non-rheumatic AVD [10,11,12,13] and is widely interpreted to represent a secondary finding of inflammation, typically due to atherosclerosis [14,15]. However, children without atherosclerosis develop AVD, and the potential use of 3-Hydroxy-3-Methylglutaryl coenzyme-A reductase inhibitors (statins), an effective therapy for atherosclerosis, to treat AVD has shown no effect on the progression of AVD or the need for aortic valve replacement [16]. The potential role of aberrant angiogenesis in early-onset AVD pathogenesis is unknown.

Elastic fiber abnormalities have long been identified as a central finding of AVD, including markedly diminished elastic fibers that are fragmented and dispersed throughout the valve cusp layers [17,18,19]. AVD in pediatric patients is characterized by similar extracellular matrix (ECM) disorganization, including elastic fiber fragmentation (EFF), but without inflammation or calcification [20,21]. Both Williams (OMIM#194050) and Marfan (OMIM#154700) syndromes, associated with mutations in the elastic fiber genes elastin (ELN) and fibrillin-1 (FBN1), respectively, are associated with valve malformation and disease in 20–45% of cases [22,23]. The histopathology abnormalities of valves in these genetic syndromes are distinct, but each overlap with non-syndromic AVD [23,24]. Interestingly, it has been shown that intact elastic fibers are angiostatic, and elastic fiber fragments are angiogenic [25,26], consistent with a potential role for EFF in the manifestation of neovascularization in AVD; however, the relationship between EFF and aberrant angiogenesis in the context of AVD is unknown.

The anatomy of the mature aortic valve is complex. There are normally three semilunar cusps hinged to a crown-shaped collagenous annulus within the aortic root [27,28]. The cusp extracellular matrix (ECM) organization is trilaminar (fibrosa, spongiosa, ventricularis) with elastic fibers radially organized as filaments in the ventricularis layer [29]. Elastic fibers consist of multiple components, including elastin, fibrillin-1, emilin, and fibulins that are coordinately expressed throughout the valve and spatio-temporally regulated during embryogenesis to facilitate fiber assembly in the ventricularis [30,31]. The ring spongiosa is a subsection of the spongiosa layer located at the interface between the annulus and cusp that functions as the hinge of the valve and consists of proteoglycans and elastic fiber components [29,32]. Little is known about the potential role of the annulus and ring spongiosa regions in AVD.

The objective of this study was to examine the impact of EFF in AVD progression. We hypothesized that early AVD would be characterized by angiogenesis, which is associated with EFF. Our findings identified EFF and aberrant angiogenesis in early-onset AVD, establishing the role of elastic fiber dysregulation in AVD pathogenesis preceding inflammation. These studies advance our understanding of early disease processes, potentially facilitating the identification of new pharmacologic treatments.

## 2. Materials and Methods

### 2.1. Human Valve Tissue

Aortic valve specimens were obtained from non-syndromic patients with isolated AVD undergoing aortic valve replacement (affected), and from age-matched individuals who died of non-cardiac causes (control) at the time of autopsy. AVD patients were stratified by age into early-onset (0–40 yo) and late-onset (41–85 yo) groups. Patients with a history of rheumatic heart disease or infective endocarditis were excluded. Medical records were reviewed, and aortic valve morphology and major cardiovascular comorbidities were noted, including coronary artery disease (CAD), essential systemic hypertension (HTN), diabetes mellitus (DM), thoracic aortic aneurysm (TAA), and chronic kidney disease (CKD).

These studies were approved by the Institutional Review Boards at Cincinnati Children’s Hospital Medical Center (CCHMC) and Good Samaritan Hospital (Cincinnati, Ohio). In addition, human heart specimens were obtained from pediatric patients with either Williams or Marfan syndrome from the Cardiac Registry at Children’s Hospital Boston; these studies were approved by the Institutional Committee on Clinical Investigations. For comparison purposes, whole heart specimens from age-matched patients with non-syndromic isolated AVD (affected) and non-cardiac disease (control) were obtained from the Teaching Collection at CCHMC.

### 2.2. Histochemistry

Valve tissue was processed and analyzed as previously described [20]. Movat’s modified pentachrome stain was used to examine ECM organization and alizarin red to assess calcification. Whole hearts were dissected precisely to demonstrate the aortic sinus equidistant from the adjacent commissures and the aortic valve annulus at its most proximal position. Comprehensive morphometrics were obtained in comparable sections, including tissue thickness dimensions for the ascending aorta, sinotubular junction, aortic root, and valve (hinge, proximal, distal), as well as area measurements for the annulus and ring spongiosa regions.

### 2.3. Immunohistochemistry

Antibodies directed against elastic fiber components, as well as markers of angiogenesis, inflammation, and atherosclerosis, were examined (Supplemental Material, Table S1). A universal streptavidin/biotin and diaminobenzidine detection system (Vector) was used for colorimetric detection, as previously described [20,21]. High heat sodium citrate antigen retrieval was used for pretreatment of all antibodies except elastin, which used enzymatic trypsin pretreatment. Angiogenesis was considered present if there was VEGF immunoreactivity without neovessel formation (provisional) or with neovessel formation (overt) [33]. Due to AVD tissue heterogeneity, a semi-quantitative assessment of VEGF and CD-68 expression was performed using a scale described by Alexopoulous et al. [34]. Briefly, staining was graded on a scale from 0 to 3 based on the percentage of positive cells as follows: 0–immunoreactivity in <10% of cells, 1—10–35%, 2—35–70%, and 3—>70%.

### 2.4. Transmission Electron Microscopy

Valve tissue ultrastructure was examined (Hitachi 7600, Hitachi, Shaumberg, IL, USA) on epoxy resin sections from 10% NBF fixed control, WS, and MFS specimens [20]. For visualization of collagens and elastins, sections were stained with 5% tannic acid aqueous solution, followed by 1% uranyl acetate, and counterstained with lead citrate.

### 2.5. Statistical Analysis

Student’s *t*-test or one-way ANOVA was used to compare groups. Findings are reported as the mean ± SEM. A *p* < 0.05 was considered significant.

## 3. Results

### 3.1. Study Population

Non-syndromic patients with AVD were stratified by age. Early-onset AVD specimens were obtained from patients aged 1 to 32 years (*n* = 21) and were compared with late-onset AVD specimens obtained from patients aged 44 to 85 years (*n* = 11). Both disease groups were also compared with age-matched controls (*n* = 8 each) that did not have AVD. The indication for surgery in all cases was stenosis, although some had mixed disease (stenosis and insufficiency). Bicuspid aortic valve (BAV) was more common in early-onset AVD, whereas cardiovascular comorbidities were more common in late-onset AVD (Table 1), consistent with previous findings [21,35,36]. Importantly, clinically significant atherosclerosis (CAD) was not present in any early-onset AVD case.

Aortic valve specimens from syndromic patients were obtained from WS (*n* = 6, mean age 22 months), MFS (*n* = 4, mean age 10 months), and compared with pediatric AVD (*n* = 3, mean age 12 months), and control (*n* = 3, mean age 5 months) patients. In addition to the characteristic arteriopathy (supravalvar aortic stenosis, SVAS) or aortopathy (dilated aortic root) identified in all WS and MFS cases examined, respectively, clinical evidence of AVD was documented in three out of five (one unknown) WS patients (one with BAV) and two out of four MFS patients (none with BAV).

### 3.2. Early-Onset AVD Specimens Demonstrate Aberrant Angiogenesis without Inflammation or Atherosclerosis

**Figure 1:**
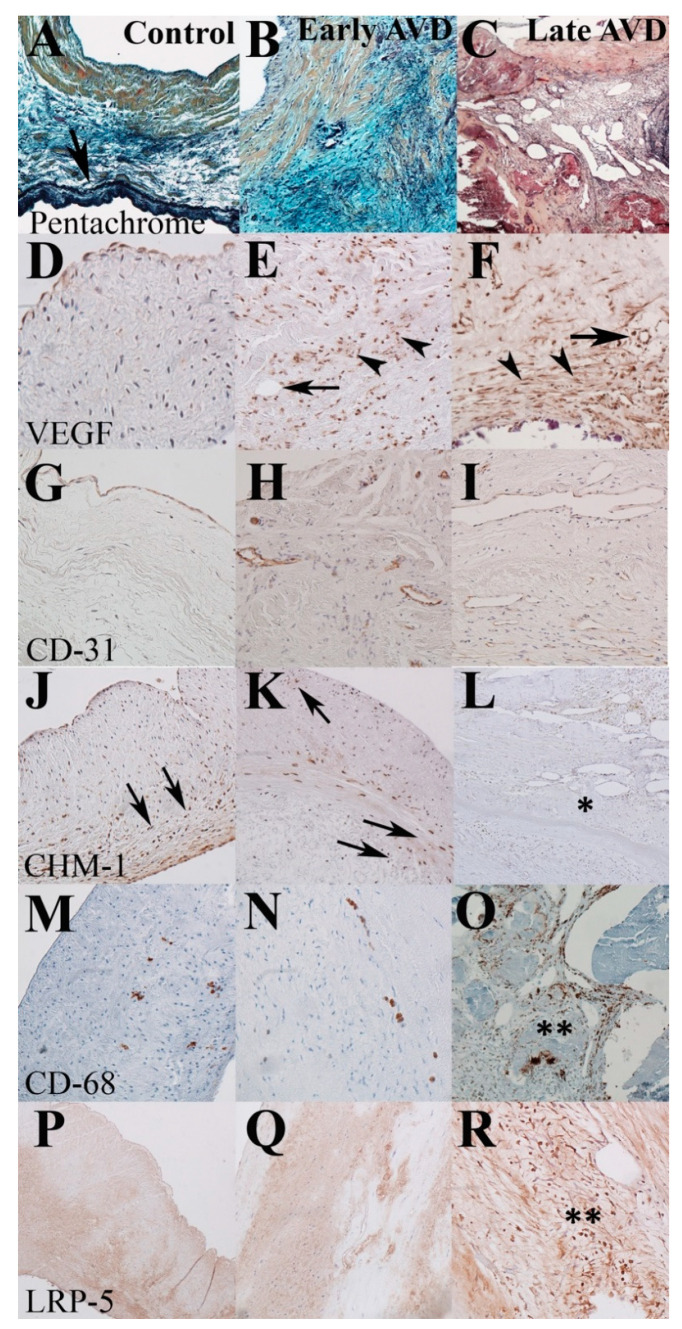
Aberrant angiogenesis in early-onset AVD precedes inflammation and atherosclerosis. Representative sections from control (left), early-onset AVD (center), and late-onset AVD (right) specimens. Normal elastic fiber structure is localized to the ventricularis layer of the cusp (arrow, **A**). Both early- and late-onset AVD are characterized by EFF and dispersion (**B**,**C**). Provisional (arrowhead) and overt (arrow) angiogenesis (**D**) is seen in early- (**E**) and late-onset (**F**) AVD, characterized in part by endothelial markers around neovessels (**H**,**I**) not observed in controls (**G**). Angiostatic CHM (**J**,**K**) is decreased in areas of angiogenesis in late-onset AVD (asterisk, **L**). Nominal CD-68 is seen in early-onset AVD (**N**), similar to control (**M**), corresponding with no atherosclerosis as demonstrated by absent LRP-5 staining (**P**,**Q**), both of which were markedly increased in late-onset AVD (double asterisks, **O**,**R**). The ventricularis cusp layer is oriented at the bottom of all panels.

Overt angiogenesis was present in 11/11 (100%) late-onset and 8/21 (38%) early-onset AVD specimens. Among the early-onset AVD subgroup, 16/21 were from pediatric patients (≤18 years old), and 4/16 (25%) showed overt angiogenesis. In both groups, neovessels were identified in all cusp layers and the annulus with an increased proportion of vessels recognized in the proximal aspect of the cusp, including the ring spongiosa; however, vessels in early-onset AVD specimens were fewer in number and smaller in diameter. Provisional angiogenesis was present to the same extent in all early- and late-onset AVD specimens but was not detected in age-matched controls. VEGF expression is increased in the interstitium heterogeneously in both early and late-onset AVD (Figure 1). CD-31 identified endothelial cells associated with neovessels but only scant interstitial cells, suggesting most VEGF-positive cells are not endothelial in origin. CD-31 positive neovessels were quantified and stratified as microvessels or arterioles (Table 2).

Chondromodulin, an angiostatic factor, is decreased in all AVD specimens, as previously described [10]. Importantly, the inflammation marker CD-68 is strongly expressed in VIC clusters spatially associated with calcific nodules in late-onset AVD but are virtually absent from the majority of early-onset specimens, similar to controls, suggesting upregulation of VEGF arose from resident cell populations. Semi-quantitative analysis for VEGF and CD-68 is reported in Table 3. Similarly, the Wnt/beta-catenin signaling marker LRP-5, which has been used to identify atherosclerosis, is absent from early-onset AVD and control specimens but is strongly expressed in late-onset AVD, as previously described [14]. Provisional and overt angiogenesis occur in early-onset AVD without clinical or histopathologic evidence of inflammation or atherosclerosis.

### 3.3. A Distinct Pattern of Elastic Fiber Components Is Associated with Early-Onset AVD Neovessels

**Figure 2:**
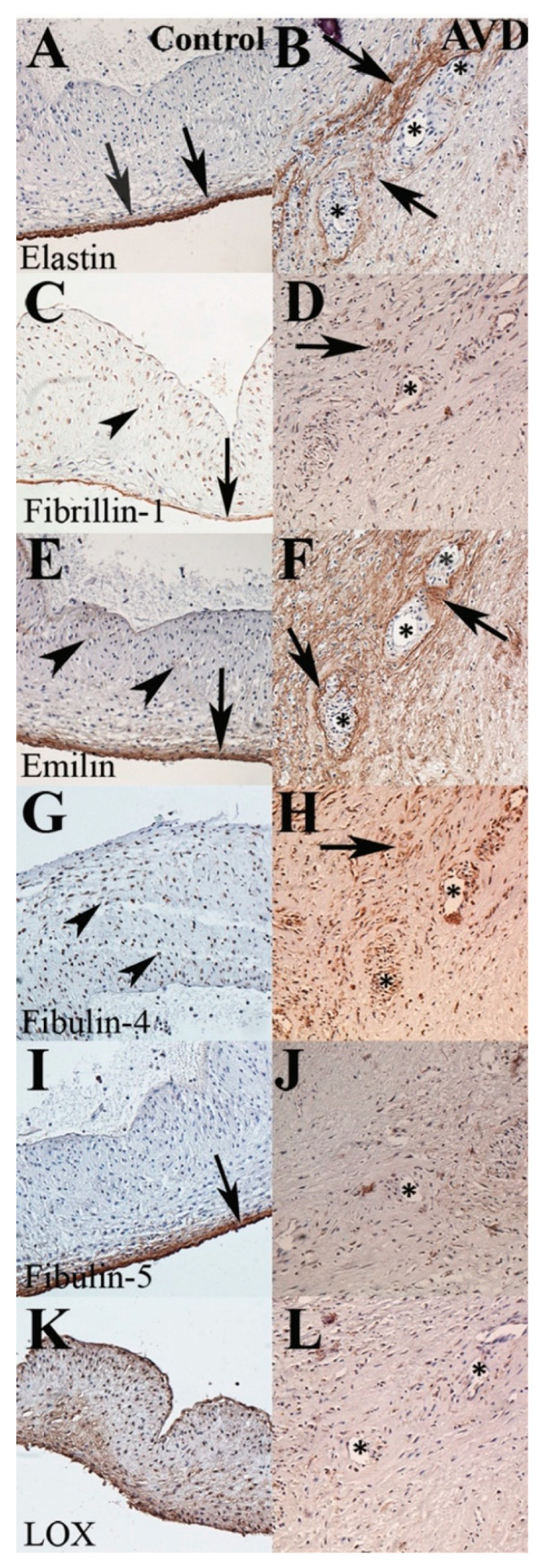
Specific elastic fiber components are associated with overt angiogenesis. Representative sections from control (left column, **A**,**C**,**E**,**G**,**I**,**K**) and early-onset AVD (right column, **B**,**D**,**F**,**H**,**J**,**L**) specimens. In control tissue, elastin and fibulin-5 are localized in the cusp ventricularis layer (arrows), but fibrillin, emilin, fibulin-4, and LOX are also expressed weakly in all layers (arrowheads). In AVD tissue, elastin, fibrillin, emilin, and fibulin-4 are associated with neovessels (asterisk, **B**,**D**,**F**,**H**), whereas fibulin-5 and LOX are not (**J**,**L**).

In early-onset AVD, elastic fibers are decreased, fragmented, and dispersed in general, as previously reported in late-onset AVD [17]; however, the distribution of specific elastic fiber components is distinct (Figure 2). Elastin is present in the fragments surrounding neovessels, whereas fibrillin is present in the lining of the neovessels but not in the area around them. Emilin, an elastic fiber glycoprotein that binds tropoelastin to the microfibril, is increased in all layers and present predominantly in the area surrounding neovessels. Fibulin-4, but not fibulin-5, is present around neovessels. Finally, LOX expression, normally present throughout the valve but preferentially in the ventricularis layer, was not changed in AVD specimens compared with controls and was not associated with neovessels. These patterns were similar in early and late-onset AVD groups. Of note, some EFF was associated with both neovessels and calcific nodules in late-onset AVD (data not shown) [37,38]. Therefore, differential expression of elastic fiber components is associated with aberrant angiogenesis in early-onset AVD.

### 3.4. WS but Not MFS Aortic Valves Demonstrate Aberrant Angiogenesis

**Figure 3:**
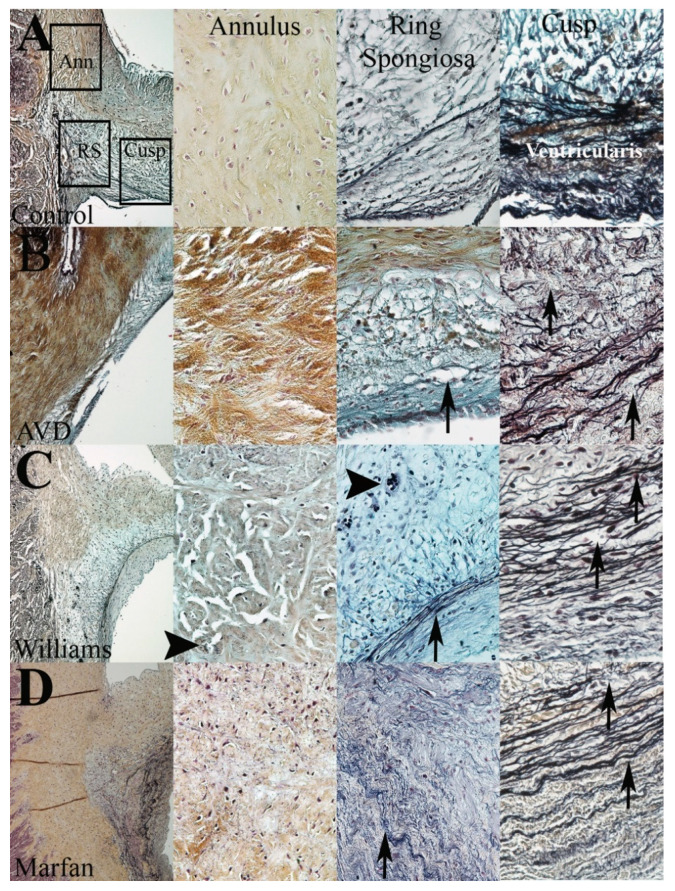
Elastic fiber dysregulation impacts regional aortic valve structure. Regional aortic valve tissue (annulus, ring spongiosa, cusp) from control (**A**), early-onset AVD (**B**), WS (**C**), and MFS (**D**) specimens demonstrate specific differences in matrix organization. EFF in WS and MFS is different (arrows, **C** vs. **D**). Neovessels are seen in WS (arrowhead, **C**) but not MFS.

To better understand the role of EFF in AVD pathogenesis, we examined the aortic valves from WS and MFS patients and compared them with early-onset AVD specimens (Figure 3). All syndromic specimens examined had histopathologic evidence of AVD. Paradoxically, elastic fiber content was markedly increased in the ventricularis layer of both WS and MFS specimens compared with non-syndromic AVD, but patterns of elastic fiber disorganization and fragmentation were different. Aortic valves from WS patients show more intra-EFF (within a bundle) and dispersion, while aortic valves from MFS patients show more inter-EFF (between bundles) and delamination. Interestingly, WS aortic valves demonstrate angiogenesis, but MFS valves do not. Elastic fiber fragments in WS were dispersed, whereas in MFS they were restricted to the ventricularis layer. In early-onset AVD, there is EFF characterized by inter- and intra-fiber fragmentation, dispersion of the fragments to all layers of the valve as well as the annulus and thinning of fibers that often remain intact with delamination. Therefore, different patterns of EFF are observed in WS and MFS aortic valves; each share characteristics seen in early-onset AVD.

### 3.5. The Aortic Root Is Composed in Part of Valve Tissue

Comprehensive morphometrics demonstrated increased valve and aorta thickness in WS specimens (Supplemental Material, Figure S1). Neither WS nor MFS aortic valves demonstrated calcification (data not shown). Neovessel formation in both WS and AVD whole heart specimens was localized to the proximal third of the cusp, the ring spongiosa, and to a limited degree the annulus, where EFF was concentrated. In WS patients, in addition to substantial collagen accumulation in the sinotubular junction (STJ), fibrillin and emilin were present within the fibrous aspect of the STJ (Supplemental Material, Figure S2). Neovessels were identified both in the fibrous STJ and the intimal aspect of the aortic media in WS. Conversely, the STJ in MFS was completely effaced, demonstrating loss of normal landmarks and no fibrous tissue. The aortic root demonstrated markedly more severe disease compared with the ascending aorta in all groups, but the patterns of histopathology were distinct by group (Supplemental Material, Figure S3). In addition to established findings [39], the WS specimens demonstrated more inter-EFF, whereas the MFS specimens demonstrated more intra-EFF. In AVD, there was less pronounced aortopathy in both the root and ascending aorta, which manifested localized areas of both inter- and intra-EFF with modest proteoglycan accumulation. Elastic fiber dysregulation contributes to both AVD and aortopathy, consistent with the clinical association, but there are different patterns of EFF by tissue type.

### 3.6. Different Elastic Fiber Defects Result in Different Types of EFF

**Figure 4:**
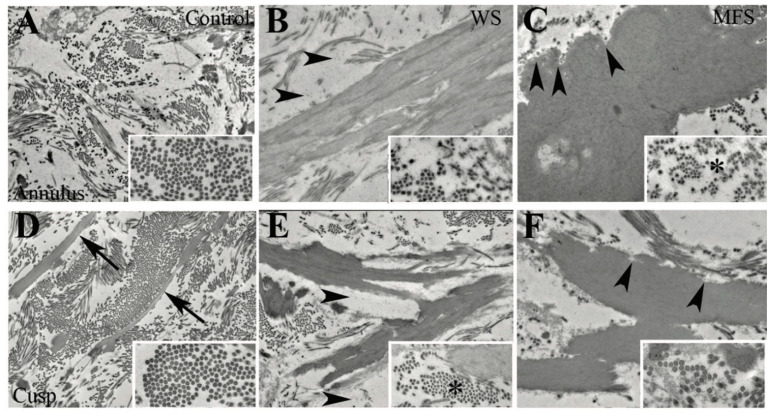
Ultrastructure of the valve annulus and cusp regions in WS and MFS. Collagen and elastic fiber organization in annulus (**A**–**C**) and cusp (**D**–**F**) regions from control (**A**,**D**), WS (**B**,**E**), and MFS (**C**,**F**) specimens. Cross sections of collagen fibril size and organization are demonstrated (insets). In control specimens, organized elastic fiber filaments are seen in the cusp (arrows) but not the annulus. WS specimens are characterized primarily by intra-EFF (**B**,**E**), whereas MFS has more inter-EFF (data not shown). There is proteoglycan accumulation in WS specimens (arrowheads, (**B**),(**E**)) and a “moth-eaten” edge appearance in MFS specimens (arrowheads, (**C**) and (**F**)). Collagen fibrils are small, irregular, and decreased in number in the cusp region of WS and the annulus region of MFS (asterisks, (**C**) and (**E**)).

An ultrastructural analysis of the aortic valve annulus and cusp regions in WS and MFS identified distinct elastic fiber abnormalities (Figure 4). In control specimens, elastic fiber components were present in the cusp ventricularis layer organized as filaments and the ring spongiosa in an unorganized fashion but were not present in the annulus. WS specimens demonstrated predominantly intra-EFF in the cusp and EFF dispersion throughout the valve, including in the annulus region, consistent with the histochemistry described above. MFS specimens demonstrated primarily inter-EFF in the cusp and EFF dispersion into the annulus but not the rest of the cusp. The elastin is characterized by a “moth-eaten” edge and a decrease in surface microfibrils, consistent with classic descriptions of fibrillin in MFS [40]. Proteoglycans were present in the annulus region of WS specimens, but not MFS or controls. Collagen fibers were disorganized in both WS and MFS groups by region compared with control specimens and previous regional descriptions [32,41]. Specifically, in WS, the collagen fiber diameters were irregular and small in the cusp region only, but in MFS, the collagen fiber diameters were irregular and small in the annulus region. Ultrastructure analysis demonstrated a spectrum of overlapping but with subtly distinct types of EFF in regional syndromic valve tissue, consistent with elastase-mediated degeneration.

## 4. Discussion

**Figure 5:**
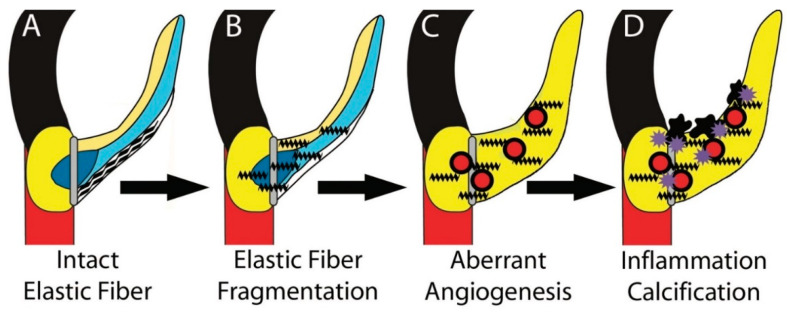
Model of early EFF and angiogenesis preceding inflammation in AVD. Intact elastic fibers are organized as filaments in the normal ventricularis cusp layer (wavy black lines, **A**). Faulty elastic fiber assembly and/or imbalance between elastases and elastase inhibitors results in EFF (jagged black lines, (**B**). EFF causes angiogenesis (red circles), increased cell proliferation, and matrix accumulation (**C**), ultimately leading to inflammation (purple stars) and calcification (black nodules, **D**).

The findings of this study identify EFF and aberrant angiogenesis as early disease processes underlying AVD preceding the manifestation of inflammation (Figure 5). These results establish a central role for elastic fiber dysregulation in early AVD pathogenesis, suggesting that faulty elastic fiber assembly and consequent elastase-mediated tissue injury contribute to disease initiation and progression. The demonstration of different types of EFF in WS and MFS aortic valves suggests that specific elastic fiber components may function in distinct ways resulting in a spectrum of disease processes. Finally, these data suggest that the annulus and ring spongiosa regions, where both shear and oscillatory stresses are concentrated and EFF and angiogenesis occur, are important in the manifestation of AVD. EFF and aberrant angiogenesis represent two disease processes that may be identifiable and modifiable early in the clinical course of AVD.

The prevailing view is that AVD is an inflammatory process, commonly associated with atherosclerosis, similar to wound healing response. In this context, tissue injury leads to inflammation and myofibroblast activation, which in turn leads to fibrosis and neovascularization [42]. Findings from previous studies support an inflammatory mechanism in the context of end-stage AVD [11,15], but it is unclear whether this represents the inciting pathology or a secondary factor that accelerates AVD progression [43,44]. Increasing evidence suggests additional mechanisms contribute to early AVD pathogenesis, including dysregulation of developmental programs [7,45,46,47,48,49], which may predispose valve tissue to inflammation. A strength of this current study is the strategy to compare early with late-onset AVD specimens [20,21] to control for the confounding effects of common comorbidities associated with inflammation in adulthood, including aging [50]. Here we have shown for the first time that EFF and aberrant angiogenesis occur in early-onset AVD in the absence of inflammation in valve tissue.

Loss of balance between elastases and elastase inhibitors has been identified as one cause of EFF, specifically the actual fragmentation of a previously normal elastic fiber in the context of both aging (physiologic) and injury (pathologic) [51,52,53]. Inflammation is characterized in part by elastolysis and EFF, which explains why these findings are considered end-stage structural findings; however, several lines of evidence suggest EFF may precede inflammation through known signaling functions [54,55], including neutrophil chemotaxis [25], identifying a potential link between EFF and pre-inflammatory processes. Furthermore, previous studies have shown that different elastic fiber fragments have different biologic functions; for example, some fragments induce calcification while others are chemo-attractants for endothelial cells [56,57]. Consistent with this idea, we have demonstrated aberrant angiogenesis in WS but not MFS aortic valves, suggesting elucidation of different elastic fiber fragment functions may identify new mechanisms underlying early AVD [58].

Gross and Kugel described the anatomy of mature heart valves in detail, noting the presence of elastic fiber components in the annulus, ring spongiosa, and fibrosa, in addition to organized elastic fiber filaments in the ventricularis [29]. In the present study, EFF and angiogenesis were demonstrated predominantly in the ring spongiosa and proximal aspect of the cusp, supporting previous observations suggesting that disease begins and worsens in the hinge area [32,43,59]. The annulus and ring spongiosa regions are especially prone to degeneration due to secondary insults, such as shear and compressive stresses [60,61,62]. Woo et al. elegantly demonstrated a complex interaction between developmental programs that predispose tissue to disease and shear stresses that trigger inflammation [63], providing an example of how these factors together contribute to AVD. Elastic fiber lamellae, the circumferential organizational unit of the ascending aorta, are disrupted in the aortic root by valve tissue due to the valve annulus’s irregular crown shape, which extends distally to the STJ. Interestingly, we observed a discrete string of cartilage-like halo cells separating the fibrous valve annulus tissue from the aortic media in the STJ (the site of obstruction in SVAS), similar in composition and location to cartilage nodules seen in the valve annulus of the elastin haploinsufficient mouse model of AVD [64,65]. The STJ in WS patients is characterized by fibrous overgrowth, in addition to dramatic thickening of the aortic media, suggesting that the left ventricular outflow tract obstruction seen in SVAS is due in part to valve disease processes. These results suggest that focused examination of the annulus and ring spongiosa regions in both human and animal research will inform our understanding of AVD progression.

Both early and late-onset AVD is characterized by EFF. Our findings suggest that some elastic fiber fragments, or specific types of EFF, result in aberrant angiogenesis, underscoring the importance of delineating elastic fiber development [66]. Since our understanding of human AVD pathogenesis is limited to observations in late-stage disease, animal models are necessary to identify mechanisms that contribute to early pathogenesis. Targeted mutagenesis of elastic fiber genes in mice has generated models characterized in part by valve phenotypes, including elastin, emilin-1, fibrillin-1, and fibulin-4 [64,67,68,69]. Likewise, genes that regulate angiogenesis, including VEGF, chondromodulin, and periostin, also play a role in valve development and disease [70,71,72]. The emilin-1 mutant mouse model of AVD demonstrates both EFF and aberrant angiogenesis [67], but the elastin haploinsufficent mouse model of AVD does not [64]. Other elastic fiber mouse models have been associated with pro-angiogenic effects or vasculature defects, but they have not reported on the potential presence of neovessels in valve tissue [55]. EFF and angiogenesis represent two early disease processes that may lead to new pharmacologic-based treatment strategies.

Presently, there are no pharmacologic-based treatment strategies for AVD. Clinical studies have identified risk factors for AVD progression, but studies examining human AVD tissue are restricted to late-stage disease. As a result, our understanding of early pathogenesis is limited. The observation that EFF and angiogenesis are early disease processes suggests that elastase and angiogenesis inhibitors represent potential new pharmacologic treatments for AVD that prevent disease progression and the need for surgery [8,73]. While nonspecific clinical risk factors for AVD have been established [36], predictive biomarkers have not been identified for early AVD progression. Aortic valve sclerosis, or thickening of the aortic valve, is a late marker of cardiovascular risk but not a specific marker for AVD [74]. The systemic effects of EFF and aberrant angiogenesis in valve tissue may provide these specific biomarkers, such as an increase in specific types of urine elastin degradation products. Finally, a better understanding of EFF-induced cell-matrix perturbations may inform the search for durable valve bioprostheses [75].

## Figures and Tables

**Table 1:** Non-syndromic AVD study population demographics and comorbidities.

	Early-Onset AVD	Late-Onset AVD	*p*
N	21	11	-
Mean Age (Range)	15 (1, 32)	67 (44, 85)	-
Male (%)	81	73	NS
BAV (%)	48	27	0.08
CAD (%)	0	45	<0.003
HT (%)	10	55	<0.002
TAA (%)	10	18	NS
DM (%)	5	36	0.08
CKD (%)	0	9	<0.001

AVD, aortic valve disease; BAV, bicuspid aortic valve; CAD, coronary artery disease; CKD, chronic kidney disease; DM, diabetes mellitus; HT, systemic hypertension; NS, not significant; TAA, thoracic aortic aneurysm.

**Table 2:** CD-31 neovessel quantification and stratification.

	Microvessels	Arterioles	Total (Vessels/10×)
Control	0	0	0
Early AVD	0.7	0.2	0.9
Late AVD	3.7	1.9	5.6

**Table 3:** VIC relative expression of angiogenesis and inflammation.

		VEGF-A			CD-68	
Score	Control	Early AVD	Late AVD	Control	Early AVD	Late AVD
**0** (0–10% + cells)	100%	0%	18%	100%	73%	20%
**1** (10–35% + cells)	0%	21%	27%	0%	18%	40%
**2** (35–70% + cells)	0%	57%	36%	0%	9%	40%
**3** (>70% + cells)	0%	21%	18%	0%	0%	0%
Mean (SD)	0 ± 0	2.0 ± 0.7 *	1.6 ± 1.0 *	0 ± 0	0.4 ± 0.7	1.3 ± 0.8 *^,#^

* *p* < 0.05 vs. Control; ^#^ *p* < 0.05 vs. Early AVD.

## Data Availability

The data presented in this study are available on request from the corresponding author.

## Supplementary Materials

The following are available online at https://www.mdpi.com/article/10.3390/jcdd8070075/s1, Table S1: Specifications of primary antibodies, Figure S1: Regional aortic valve and aorta pathology in syndromic and non-syndromic AVD, Figure S2: Valve tissue contributes to the sinotubular junction (STJ) narrowing in the WS aorta (AO), and Figure S3: Aortic root histopathology is more severe and distinct from ascending aorta pathology in syndromic and nonsyndromic AVD.
