# Supplementary material for: Early Aberrant Angiogenesis Due to Elastic Fiber Fragmentation in Aortic Valve Disease
Source: J Cardiovasc Dev Dis. 2021 Jun 25;8(7):75. doi: 10.3390/jcdd8070075 (PMC8303641; doi:10.3390/jcdd8070075)
Supplement: Supplementary file 1 [file jcdd-08-00075-s001.zip › jcdd-1230519-supplementary.pdf]

Supplemental Information

Supplemental Tables

Table S1. Specifications of primary antibodies

Protein	Marker	Host and Type	Source	Dilution
Elastin	Elastic Fiber	Mouse monoclonal	Sigma-Aldrich	1:500
Fibrillin-1	Elastic Fiber	Rabbit polyclonal	Abcam	1:50
Emilin-1	Elastic Fiber	Rabbit polyclonal	Sigma-Aldrich	1:50
Fibulin-4	Elastic Fiber	Rabbit polyclonal	Novus Biologicals	1:50
Fibulin-5	Elastic Fiber	Mouse monoclonal	Abcam	1:500
Lysyl Oxidase	Elastic Fiber	Rabbit polyclonal	Lifespan Bioscience	1:75
VEGF-A	Angiogenesis	Rabbit polyclonal	Santa Cruz, 152	1:50
Chondromodulin	Angiostasis	Rabbit polyclonal	Lifespan Bioscience	1:50
CD-31	Endothelial cells	Rabbit polyclonal	Abcam	1:100
CD-68	Inflammation	Rabbit polyclonal	Ventana	prediluted
LRP-5	Atherosclerosis	Rabbit polyclonal	Biovision	1:50

Supplemental Figures

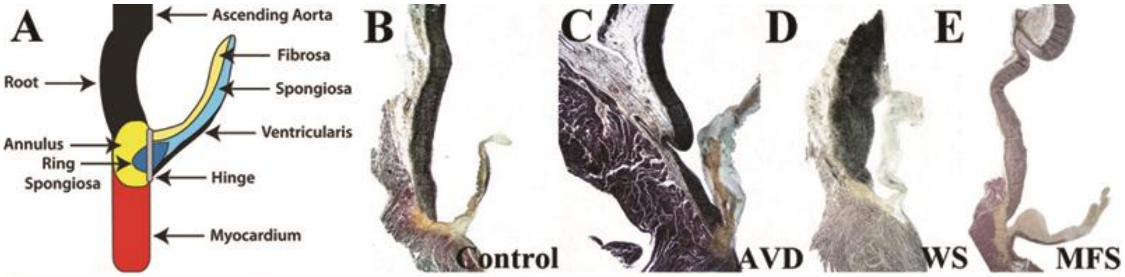

Regional Aortic Valve and Aorta Morphometrics in Syndromic and Nonsyndromic Aortic Valve Disease

	Control	AVD	Williams	Marfan
**Valve Hinge thickness (µm)**	611 ± 42	426 ± 13*	924 ± 62*	462 ± 62*
**Proximal cusp thickness (µm)**	274 ± 63	731± 14*	471 ± 69*	620 ± 48*
**Distal cusp thickness (µm)**	340 ± 64	548 ± 75*	484 ± 69	248 ± 30
**Annulus area (µm2)**	423006 ± 132425	703107 ± 206783	377256 ± 38923	238741 ± 48954
**Ring Spongiosa area (µm2)**	48252 ± 39206	22338 ± 1866*	73568 ± 12886	45928 ± 6298
**Aortic Root thickness (µm)**	430 ± 40	394 ± 70	749 ± 53*	346 ± 43
**Ascending Aorta thickness (µm)**	444 ± 7	553 ± 53	931 ± 86*	487 ± 11*

* $p < 0.05$

Figure S1. Regional aortic valve and aorta pathology in syndromic and non-syndromic AVD. A model of unaffected aortic root anatomy shows the annulus and ring spongiosa regions in a longitudinal plane evenly separating a sinus of Valsalva (A). Low magnification images (Scale 1X) of whole heart specimens demonstrate gross differences between early AVD (C), WS (D) and MFS (E) specimens when compared to Control (B). The aortic root and ascending aorta measurements are restricted to the medial layer. Related morphometrics are shown in the Table.

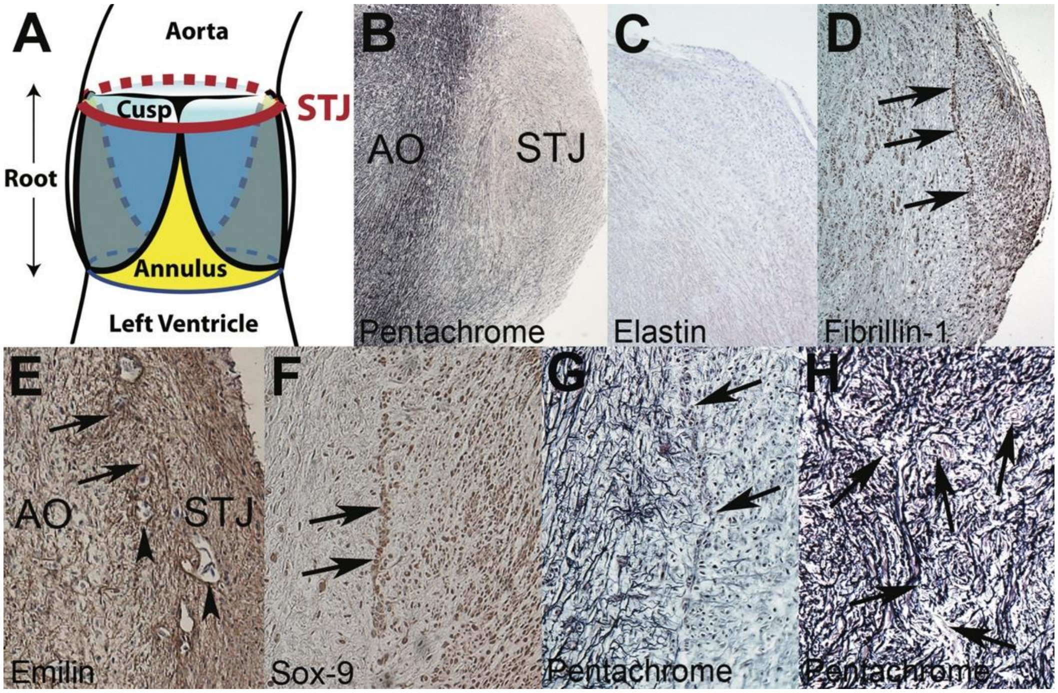

Figure S2. Valve tissue contributes to the sinotubular junction (STJ) narrowing in the WS aorta (AO). The complex anatomy of the aortic valve and aortic root is shown in panel A. The STJ is fibrous (B), consistent with valve tissue, and characterized by weak elastin expression (C) and strong fibrillin (D) and emilin (E) expression. There is a string of cartilage-like halo cells at the boundary of fibrous and artery tissues (arrows, E,F,G), which is characterized by positive Sox-9 expression (F). There is neovessel formation both in the fibrous STJ (arrowheads, E) and the proximate aortic media (arrows, H).

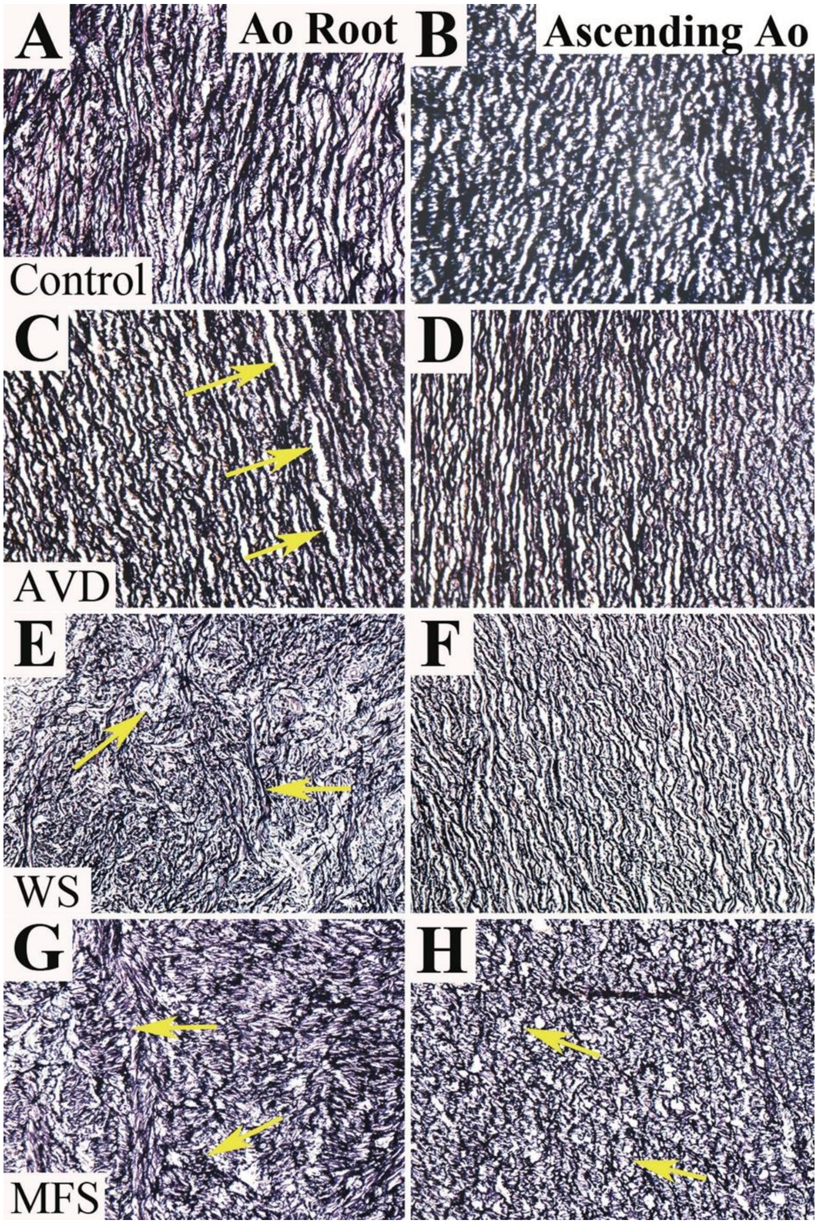

Figure S3. Aortic root histopathology is more severe and distinct from ascending aorta pathology in syndromic and nonsyndromic AVD. The aortic root (A,C,E,G) and ascending aorta (B,D,F,H) is shown in control (A,B), early AVD (C,D), WS (E,F), and MFS (G,H). MFS aortic roots are more severely affected (G vs. H), characterized by disrupted intra-EFF. In contrast, WS and AVD aortic root tissue is characterized by disrupted inter-EFF.
